# Comparative Genomic Analysis of Two Novel Sporadic Shiga Toxin-Producing *Escherichia coli* O104:H4 Strains Isolated 2011 in Germany

**DOI:** 10.1371/journal.pone.0122074

**Published:** 2015-04-02

**Authors:** Erhard Tietze, Piotr Wojciech Dabrowski, Rita Prager, Aleksandar Radonic, Angelika Fruth, Philipp Auraß, Andreas Nitsche, Martin Mielke, Antje Flieger

**Affiliations:** 1 Department of Infectious Diseases, Division of Enteropathogenic Bacteria and *Legionella*, National Reference Centre for *Salmonella* and other Bacterial Enteric Pathogens, Wernigerode Branch of Robert Koch-Institute, Berlin, Germany; 2 Centre for Biological Threats and Special Pathogens, Robert Koch-Institute, Berlin, Germany; 3 Department of Infectious Diseases, Robert Koch-Institute, Berlin, Germany; Institut National de la Recherche Agronomique, FRANCE

## Abstract

A large outbreak of gastrointestinal disease occurred in 2011 in Germany which resulted in almost 4000 patients with acute gastroenteritis or hemorrhagic colitis, 855 cases of a hemolytic uremic syndrome and 53 deaths. The pathogen was an uncommon, multiresistant *Escherichia coli* strain of serotype O104:H4 which expressed a Shiga toxin characteristic of enterohemorrhagic *E*. *coli* and in addition virulence factors common to enteroaggregative *E*. *coli*. During post-epidemic surveillance of Shiga toxin-producing *E*. *coli* (STEC) all but two of O104:H4 isolates were indistinguishable from the epidemic strain. Here we describe two novel STEC O104:H4 strains isolated in close spatiotemporal proximity to the outbreak which show a virulence gene panel, a Shiga toxin-mediated cytotoxicity towards Vero cells and aggregative adherence to Hep-2 cells comparable to the outbreak strain. They differ however both from the epidemic strain and from each other, by their antibiotic resistance phenotypes and some other features as determined by routine epidemiological subtyping methods. Whole genome sequencing of these two strains, of ten outbreak strain isolates originating from different time points of the outbreak and of one historical sporadic EHEC O104:H4 isolate was performed. Sequence analysis revealed a clear phylogenetic distance between the two variant strains and the outbreak strain finally identifying them as epidemiologically unrelated isolates from sporadic cases. These findings add to the knowledge about this emerging pathogen, illustrating a certain diversity within the bacterial core genome as well as loss and gain of accessory elements. Our results do also support the view that distinct new variants of STEC O104:H4 repeatedly might originate from yet unknown reservoirs, rather than that there would be a continuous diversification of a single epidemic strain established and circulating in Germany after the large outbreak in 2011.

## Introduction

During a large outbreak of gastrointestinal disease 2011 in Germany, almost 4000 people contracted acute gastroenteritis or hemorrhagic colitis, 855 cases developed a hemolytic uremic syndrome (HUS) and 53 patients died [1]. Simultaneously, a smaller outbreak occurred in France [2, 3]. The pathogen in both of the outbreaks was an uncommon, multiresistant Shiga-toxinogenic *Escherichia coli* (STEC) strain of serotype O104:H4. PCR-based virulence gene analysis of the outbreak strain revealed a combined pattern of virulence properties inherent to two different *E*. *coli* pathovars [4–6]. The production of a Shiga toxin (Stx) is a signature property of enterohemorrhagic *E*. *coli* (EHEC). In addition this strain contained a plasmid which encodes the aggregative adherence-mediating fimbriae (AAF) characteristic of enteroaggregative *E*. *coli* (EAEC). Immediate extensive characterization of the outbreak strain by whole genome sequencing confirmed its hybrid pathovar type [7–11]. Routine epidemiological typing at the German National Reference Center for *Salmonella* and other Bacterial Enteric Pathogens (NRC) of hundreds of STEC O104:H4 isolates during the outbreak using pulsed-field gel electrophoresis (PFGE) after macrorestriction, plasmid profile analysis, virulence gene profiling and antibiotic susceptibility testing did not recognize any variation [12].

On July 26^th^ 2011 the German national public health authority, the Robert Koch Institute, declared the German outbreak to be over, dating the begin on May 5^th^ and the end on July 4^th^ with the peak on May 22^nd^ [13]. During post-epidemic surveillance of STEC-caused disease by the German NRC, all but two of O104:H4 isolates remained indistinguishable from the epidemic strain. Here we describe two novel post-epidemic STEC O104:H4 strains isolated in close spatiotemporal proximity to the outbreak. These isolates differed both, from the epidemic strain and from each other by features as determined by the routine epidemiological typing methods to such an extent, that they were not considered to be isolates of the O104:H4 outbreak strain but most likely isolates from sporadic cases. Whole genome sequencing of the two sporadic strains, of ten outbreak strain isolates originating from different cases and time points of the outbreak and of one historical sporadic EHEC O104:H4 isolate was performed. Analyses and comparison with other published sequences of EHEC O104:H4 outbreak and sporadic isolates clearly confirmed a phylogenetic relatedness but epidemiological distance between the sporadic strains and the outbreak strain.

## Materials and Methods

### *Escherichia coli* O104:H4 strains

All *E*. *coli* strains under investigation (Table 1) were isolated from enriched cultures of coliform bacteria and serotyped at the NRC. Isolates obtained during and shortly after the outbreak period are from infections between May and August 2011. Strain 11–02027 is the index strain provided for reference by the Robert Koch-Institute in May 2011. PCR-based virulence gene profiling revealed the strain Shiga toxin gene *stx*_1_ negative, *stx*_2a_ positive, intimin gene *eae* negative, ABC-transporter protein gene *aatA* positive, master regulator gene of virulence-plasmid genes *aggR* positive, secreted protein dispersin gene *aap* positive, AAF-fimbrial operon genes *aggA*/type I and *aggC*/type I positive, enteroaggregative *E*. *coli* heat-stable enterotoxin gene *astA* negative [5, 12]. Nine more typical outbreak isolates from clinical cases (Table 1) which were indistinguishable from 11–02027 by routine epidemiological typing methods such as virulence gene profiling, plasmid profile analysis, antibiotic susceptibility testing and PFGE after macrorestriction were selected for whole genome sequencing. Isolates 11–06681 and 11–07153 attracted attention during post epidemic routine surveillance as they share the virulence gene profile with the outbreak strain but differed from the outbreak strain and from each other with respect to the antibiogram (Table 1). The historical O104:H4 isolate 01–09591 originates from the same patient sample as the well characterized EHEC strain HUSEC041 [7, 14]. Isolated in 2001, it is considered epidemiologically unrelated to the 2011 outbreak strain and was therefore included in our investigations for comparison.

**Table 1:** Origin of the STEC O104:H4 isolates under investigation and characteristics as determined by standard subtyping methods.

Strains ^a)^	Antibiotic resistance profile ^b)^	PFGE profile ^c)^	Plasmid profile ^d)^ [kilo basepairs]	date of isolation / clinical background / note
11–02027	Tc Sm Sxt Ap Nal Ceph	type I	88; 75; 1.5	May 19^th^, 2011 / bloody diarrhoea, HUS / outbreak index isolate
11–02058, 11–02135, 11–03424, 11–03944, 11–04083, 11–06601, 11–06782, 11–06811, 11–06837	Tc Sm Sxt Ap Nal Ceph	type I	88; 75; 1.5	outbreak isolates obtained between May 20^th^ and August 12^th^, 2011 / bloody diarrhoea, HUS, post-diarrhoeal shedding
11–06681	Tc Sm Sxt Ap Nal	type II	95; 75; 53; 36	August 1^st^, 2011 / asymptomatic / sporadic isolate
11–07153	Tc Sm Sxt Ap Nal Cm	type III	75; 60	August 22^nd^, 2011 / bloody diarrhoea / sporadic isolate

^a)^ All isolates share the same virulence gene PCR profile: *stx*_1_ negative, *stx*_2a_ positive, *eaeA* negative, *aatA* positive, *aggR* positive, *aap* positive, *aggA*/type I and *aggC*/type I positive, *astA* negative

^b)^ Antibiotic resistance phenotypes: Tc—tetracyclines, Sm—streptomycin, Sxt—trimethoprim/ sulphonamides, Ap—ampicillin, Nal—nalidixic acid: Nal, Ceph—cephalosporins, Cm—chloramphenicol.

^c)^ see Fig 1

^d)^ see Fig 2

### Standard diagnostic procedures

Conventional serotyping, a broth microdilution method for testing susceptibility against antibiotics, PCR for virulence gene profiling, macrorestriction analysis using enzyme XbaI, pulsed-field gel electrophoresis (PFGE), plasmid profiling and multi locus sequence typing (MLST) assigning alleles and sequence type in accordance with the *E*. *coli* MLST database [16] were carried out as described elsewhere [5, 15, 17].

#### Cytotoxicity Assay

Toxicity towards Vero cells was determined as described previously [17]. Briefly, strains were grown to exponential phase in TSB (Difco), then diluted 1:100 in 5ml TSB and incubated for 20h at 37°C with agitation (180rpm). Next, 100ul of 8-fold to 512-fold DMEM (GE Healthcare) diluted cell free culture supernatants of the TSB-grown strains were added to washed confluent Vero cell monolayers in 100ul DMEM/10% FCS in 96 well plates in triplicates. For each experiment fresh culture supernatants were produced and equal growth of the bacterial cultures was confirmed by OD600 readings. After 48h of incubation at 37°C, supernatants were analyzed for LDH release by means of the CytoTox96 Non-Radioactive Cytotoxicity Assay (Promega) according to the manufacturer’s protocol.

#### Adherence to Hep-2 cells

The Hep-2 cell adherence assay was performed as previously described [17]. Briefly, bacteria were grown to exponential growth phase in TSB (Difco), then inoculated 1:100 in 5ml TSB containing 1% D-mannose and incubated for 20h statically at 37°C. Equal growth of the cultures was confirmed by reading OD600. Hep-2 cells, grown to 70 to 90% optical confluence in 24 well plates (in DMEM/10% FCS, GE Healthcare), were washed with PBS nd the medium was replaced with DMEM containing 1% D-mannose. Subsequently, 40ul of the bacterial cultures were added per well. After 3h of incubation, cells were washed three times with PBS, followed by fixation in ice-cold 70% ethanol on ice for 15min. Next, samples were stained with Giemsa staining solution (1/20 diluted 0.4% stock solution, diluted in PBS) for 20min at room temperature. Samples were then rinsed with water, air dried, and mounted for microscopy at 600-fold magnification on a Nikon Eclipse inverted microscope.

#### Library preparation and genome sequencing

Genomic DNA was prepared using the Qiagen DNeasy Blood & Tissue-Kit according to the instructions of the supplier. One μg of DNA as determined with the Qubit-dsDNA BR assay and instrument (Invitrogen) was fragmented using a Covaris S2 instrument (Covaris Ltd., Woodingdean Brighton, UK). The fragmented DNA was used to generate libraries for 454 sequencing utilising the GS Rapid Library Prep Kit (Roche Diagnostics, Mannheim, Germany) following the manufacturer’s instructions. All libraries contained the genomic sequences for amplification, the sequencing primer binding sequence and multiplex identifier sequences for multiplexing. The libraries were amplified utilising the GS Titanium MV emPCR v2 Kit (Roche Diagnostics, Mannheim, Germany). Sequencing was based on the 454-pyrosequencing chemistry from Roche. The Roche FLX+ instrument was used in combination with the GS FLX Titanium Sequencing Kit XL+ chemistry (Roche Diagnostics, Mannheim, Germany). Base calling was performed by the instrument’s software.

The raw data are deposited in the single read archive at GeneBank and available under the project numbers SAMN03168461 (11–02027), SAMN03174137 (11–02058), SAMN03168462 (11–02135), SAMN03168463 (11–03424), SAMN03168465 (11–03944), SAMN03168466 (11–04083), SAMN03168467 (11–06601), SAMN03168471 (11–06681), SAMN03168468 (11–06782), SAMN03168469 (11–06811), SAMN03168470 (11–06837), SAMN03168472 (11–07153), SAMN03174138 (01–09591).

#### Genome sequence analysis, single nucleotide polymorphism prediction and phylogenetic analysis

The reads for each of the 13 isolates were mapped to the genome of the *E*. *coli* O104:H4 strain TY2482 (accession number NZ_AFVR01000000) [8,9] using Bowtie2 [18]. The mappings of the reads from the outbreak index isolate 11–02027 and those from the two variant isolates 11–06681 and 11–07153 to TY2482 were further used for a basic analysis of the coverage of chromosomal coding sequences. The number of bases covered in each of the (putative) coding sequences annotated for the TY2482 chromosome [19] was recorded for each of these strains (S2 Table).

Single nucleotide polymorphisms (SNPs) were called using the GATK pipeline [20]. For this analysis we included the three finished sequences published in 2012 by Ahmed et al. [21], one from another outbreak isolate (2011C-3493, accession number NC_018658), and two others (2009EL-2050, NC_018650 and 2009EL-2071, NC_018661) from independent isolates discovered in Georgia in 2009. Moreover, the sequences of two other historical STEC O104:H4 isolates from France (Ec04-8351, AFRL01000000 and Ec09-7901, AFRK0100000) and of five more recent STEC O104:H4 isolates from sporadic cases in France (Ec11-9450, AGWF01000000; Ec12-0465, AIPQ01000000; Ec12-0466, AIPR01000000; Ec11-9941, AGWH01000000 and Ec11-9990, AGWG01000000) as published by Grad et al. [22, 23] were also included in the analysis. Using custom python scripts, positions for which no sequence information was present in at least one of the strains (i.e. where no reads from at least one of the strains mapped to the reference) were discarded. Furthermore, all positions at which the sequence information was identical for all strains (i.e. none of the strains contained a SNP or all of the strains contained the same SNP) were discarded. Conceptually, this approach results in a collection of SNPs identical to what would be obtained by creating a whole-genome alignment of all strains and discarding all identical sites. However, it allows to avoid the time-consuming and complex process of finishing the whole genome of each strain. From the resulting collection of 662 SNPs we then removed all those located inside of any of the eight chromosomal regions which were identified as prophages or prophage-related by Ahmed et al. and Grad et al. [21, 23] or obviously inside of potentially repeated DNA elements such as tRNA-genes, rRNA-operons, transposons, IS sequences etc. An alignment containing only the bases at the remaining 224 positions was then created (files S1 Dataset for SNP positions and S2 Dataset for sequences) and a phylogenetic tree was calculated using MrBayes [24] with the HKY85 substitution model and a chain length of 1,100,000. The historical isolate 01–09591 was used as outgroup.

All reads from the samples 11–02027, 11–06681 and 11–07153 were also mapped against a total of 11 reference sequences of plasmids present in strains TY2482 (three plasmids) [8, 9], 2011C-3493 (three plasmids), 2009EL-2050 (three plasmids) and 2009EL-2071 (two plasmids) [21]. Mapping was performed using bowtie2 [18]. The percentage of coverage of the respective reference sequences is recorded in supplementary file S3 Table.

From the single reads, contigs for use in homology analyses were generated for each of our isolates. Reads were both mapped to the TY-2482 genome using GS Reference Mapper 2.4 (Roche) and assembled de novo using MIRA 3.2 [25, 26]. The contigs resulting from both approaches were then assembled together using Geneious 7.1 [27] to create a set of longer contigs for each strain. The tools provided by the Geneious software were also used for phylogenetic analysis and further DNA manipulations such as extraction of DNA sequences for homology analyses utilizing the bl2seq facility [28] at the NCBI BLAST homepage [29].

## Results

### Conventional subtyping

#### Identification of two novel isolates of STEC O104:H4 from sporadic cases

**Fig 1:**
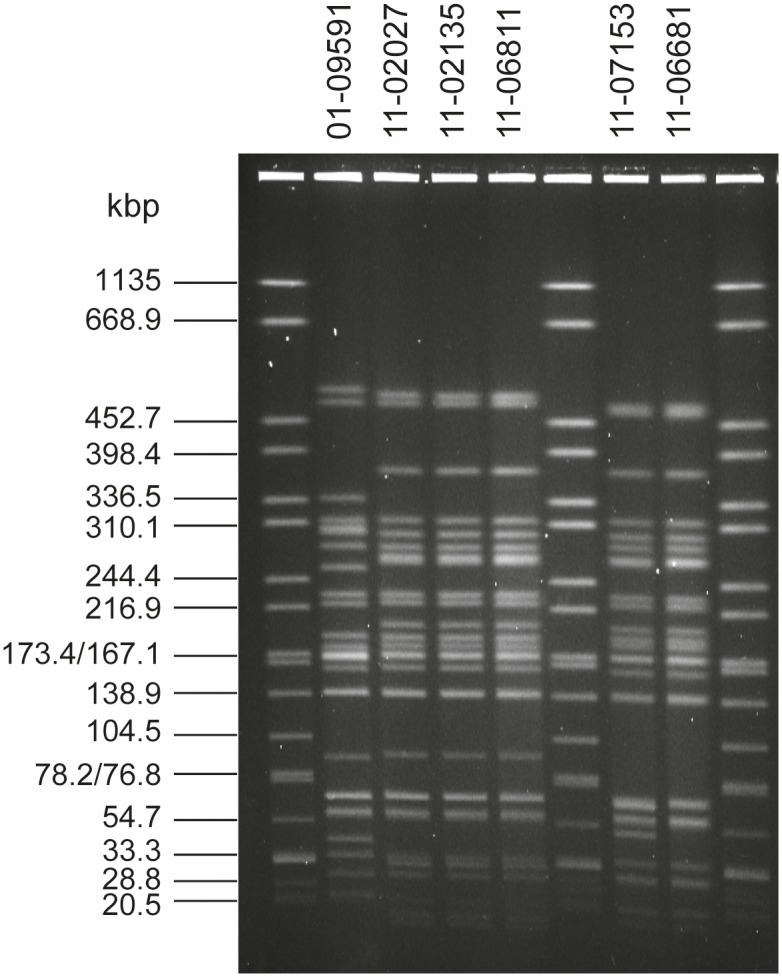
The *Xba*I macrorestriction patterns of the two sporadic STEC O104:H4 strains 11–06681 and 11–07153 are distinct from outbreak strain isolates and from the historical strain. Lanes 1, 6, 9: molecular weight standard *Salmonella* serotype Braenderup strain H9812; Lane 2: historical O104:H4 isolate 01–09591; Lanes 3, 4, 5: independent isolates of the epidemic strain (11–02027, 11–02135, 11–06811); Lanes 7, 8: variant isolates 11–07153 and 11–06681.

**Fig 2:**
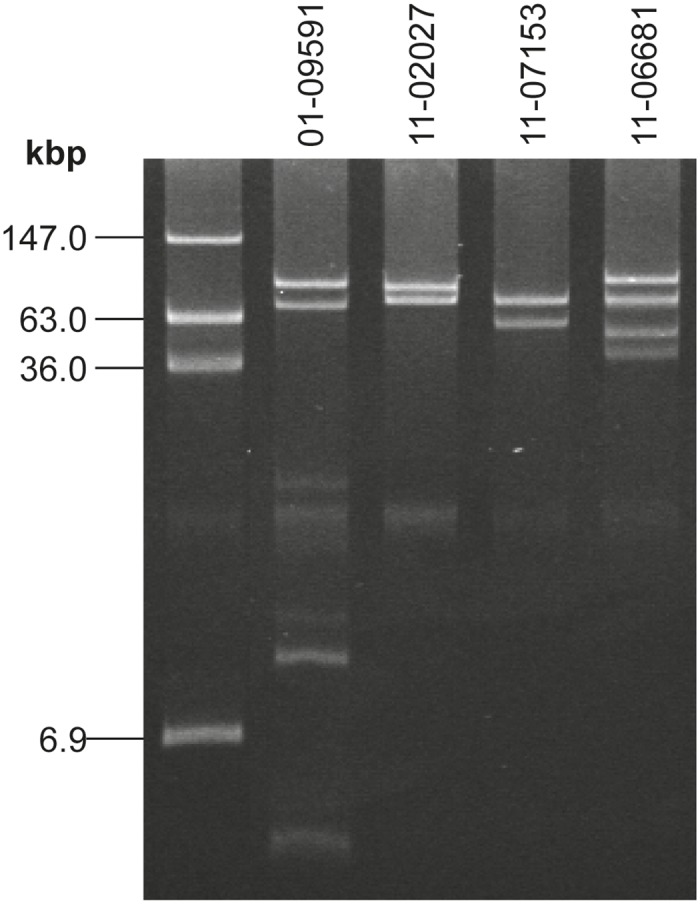
The plasmid profiles of the two sporadic STEC O104:H4 strains 11–06681 and 11–07153 are distinct from outbreak strain isolates and from the historical strain as well as from each other. Lane 1: molecular weight reference *E*. *coli* 39R862; Lane 2: historical O104:H4 isolate 01–09591; Lane 3: index isolate of the epidemic strain 11–02027—another plasmid of 1.5kbp is not visible here; Lanes 4, 5: variant isolates 11–07153 and 11–06681. A small plasmid of 1.5kbp present in 11–02027 as well as in 11–07153 and 11–06681 is not visible here (but see S3 Table).

Several hundred STEC O104:H4 isolates obtained in Germany from May to end of August 2011 were further subtyped at the German NRC [13]. Typing by conventional methods such as PFGE after macrorestriction, virulence gene profiling, plasmid profile analysis and antibiotic susceptibility testing revealed the vast majority of these isolates indistinguishable from the prototypic outbreak isolate 11–02027 (Table 1). However, during post-epidemic surveillance, two STEC O104:H4 isolates were detected which shared the virulence gene profile and the MLST sequence type ST678 with the epidemic strain but differed by several features as determined by the routine typing methods (see 11–06681 and 11–07153 in Table 1). The antibiograms of these two strains differed from that of the epidemic strain by a lack of the cephalosporin resistance phenotype. In addition, strain 11–07153 showed a chloramphenicol resistance phenotype while neither the typical outbreak strain isolates nor isolate 11–06681 did so (Table 1). With regard to their *Xba*I-PFGE patterns, strains 11–06681 and 11–07153 differed by several bands from the epidemic strain and by at least one band from each other while the historical isolate 01–09591 (see Material and Methods section) was clearly distinct (Fig 1). Plasmid profile analysis showed obvious differences between the strains (Fig 2). A plasmid of about 75kbp present in 11–02027 was identified as the AAF encoding plasmid of the epidemic strain [17]. A plasmid of same size is also present in 11–06681 and 11–07153. Three more large plasmids present in 11–06681 and one more plasmid present in 11–07153 distinguished these strains from both the epidemic strain and the historical isolate 01–09591 (Fig 2). Overall, according to customary epidemiological typing results, strains 11–06681 and 11–07153 would be considered phylogenetically related to the outbreak strain but not as outbreak strain isolates. Rather they would be regarded as independent isolates from sporadic cases.

#### The two novel sporadic STEC O104:H4 strains and the outbreak strain show comparable degrees of cytotoxicity and aggregative adherence

**Fig 3:**
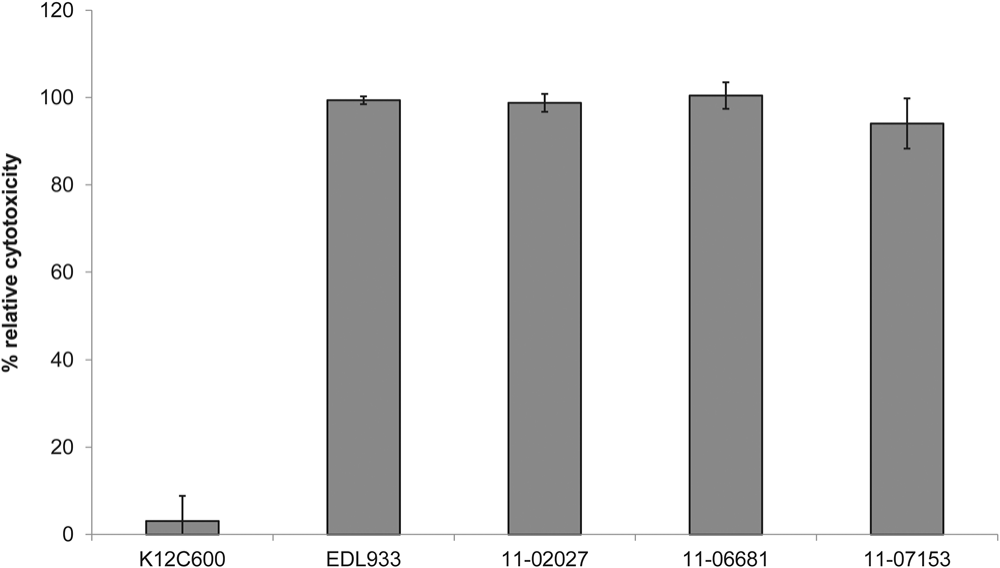
The two sporadic STEC O104:H4 strains 11–06681 and 11–07153 and the outbreak strain isolate 11–02027 show comparable levels of toxicity towards Vero cells. EHEC EDL933 served as a positive control and *E*. *coli* K12 C600 as a negative control. Toxicity of strain EDL933 as a quantitative reference was set to 100%. Shown are mean values of three independent experiments, each performed in triplicates. Bars represent means and standard deviation of three experiments. No significant difference in cytotoxicity of the STEC strains was observed.

**Fig 4:**
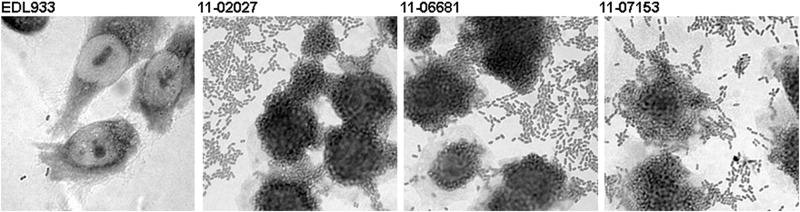
The two sporadic STEC O104:H4 strains 11–06681 and 11–07153 and the outbreak strain isolate 11–02027 show aggregative adherence as characteristic for EAEC. Assay of mannose-resistant adherence to Hep-2 cells included the EHEC O157:H7 strain EDL933 for comparison. Images were taken at 600-fold magnification.

We compared the toxicity of Stx released by the two sporadic isolates, by the outbreak strain, and by the EHEC O157:H7 strain EDL933. A quantitatively comparable level of toxicity towards Vero cells was detectable for all of the STEC strains (Fig 3). Furthermore, the sporadic strains showed the same stacked brick-like aggregative adherence pattern type as the outbreak strain which is characteristic for EAEC (Fig 4).

### Genomic analysis

#### Comparative whole genome sequence analysis of epidemic and sporadic STEC O104:H4 isolates

In order to verify the conclusions from routine epidemiological typing and to analyze their phylogenetic relatedness, comparative whole genome sequencing was performed for the two variant STEC O104:H4 strains and for the outbreak index isolate 11–02027. In addition nine independent outbreak-strain isolates and a historical STEC O104:H4 isolate from 2001 were sequenced (Table 1). For the outbreak isolate 11–02027, de novo assembly of the reads resulted in 33 contigs and for the two sporadic isolates 11–06681 and 11–07153 in 52 and 53 contigs, respectively. Mapping of the reads obtained for these strains onto the O104:H4 outbreak strain TY2482 genome covered 99.22% of this reference chromosome in the case of outbreak isolate 11–02027 and 98.21% and 98.18% with the variant strains 11–06681 and 11–07153, respectively (S1 Table). Moreover, a preliminary assessment of coverage of the chromosomal (putative) coding sequences annotated for TY2482 [19] was performed from these mappings (S2 Table). This approach revealed about 96% of the (putative) genes annotated for TY2482 present in our outbreak isolate 11–02027 genome sequence. Of these genes 2.98% were not detected in 11–06681, 2.87% were not detected in 11–07153 and 1.42% remained undetected in both variant isolates.

#### Phylogenetic analysis

**Fig 5:**
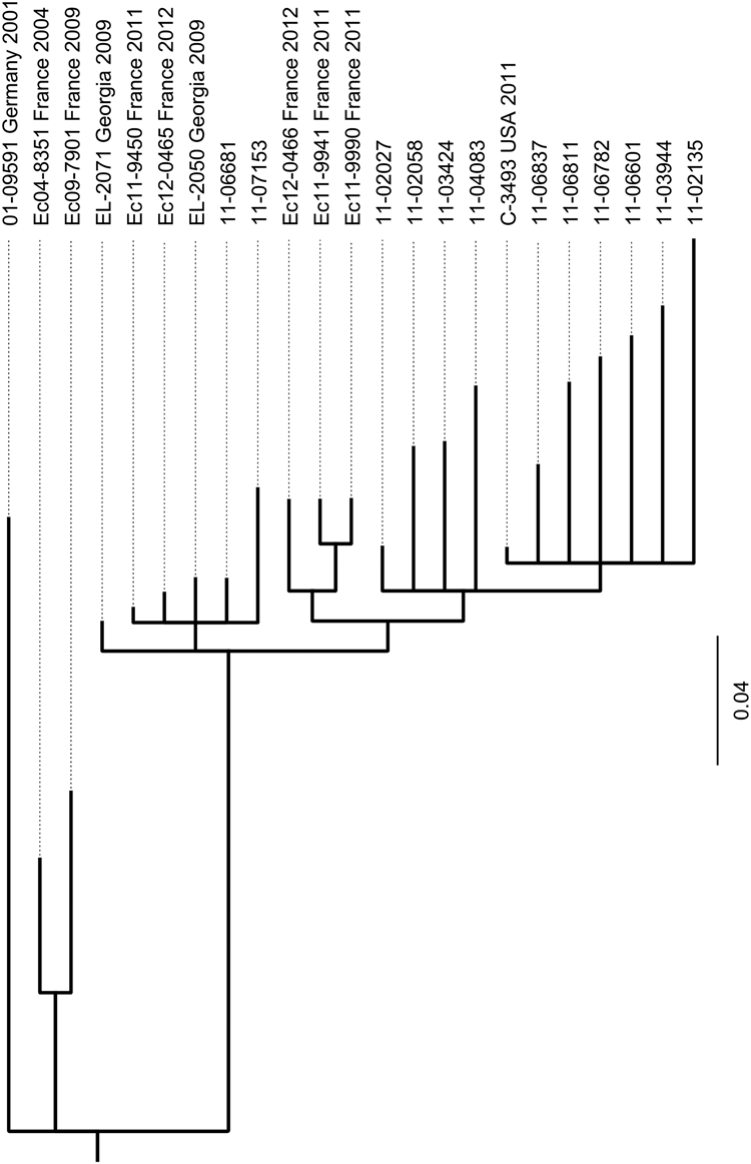
Phylogeny of the 13 STEC O104:H4 isolates under investigation and of ten more STEC O104:H4 strains based on selected SNPs in their published genome sequences assignes the two German sporadic isolates 11–06681 and 11–07153 to a clade clearly separate from the one containing the 2011 outbreak strain isolates. The tree was calculated using the MrBayes tool from the GENEIOUS software [26] and routed to the German historical isolate 01–09591 which served as outgroup. The sequences for EL-2071 and EL-2050, two sporadic isolates from cases in Georgia in 2009 and C-3493, an isolate obtained in the USA from a person with travelling history to Germany in May 2011 were published by Ahmed et al [21]. The sequences for Ec04-8351 and Ec09-7901, two historical strains from France and the sequences of Ec11-9450, Ec11-9941, Ec11-9990, Ec12-0456, Ec12-0466, five more recent sporadic isolates from France were published by Grad et al [22, 23]. The selected SNPs used for tree calculation are given in supplementary files S1 Dataset (SNP positions) and S2 Dataset (sequences).

In order to establish the phylogenetic relatedness of STEC O104:H4 strains, we included other published high quality genome sequences of STEC O104:H4 isolates in our SNP-based phylogenetic analysis. Among these are three finished sequences published in 2012 by Ahmed et al. [21], one from another outbreak isolate and two others from independent isolates discovered in Georgia in 2009. In addition the sequences of two other historical STEC O104:H4 isolates from France and of five more recent STEC O104:H4 isolates from sporadic cases in France as published by Grad et al. [22, 23] were also included in the analysis. SNPs were identified comparing these sequences and the genome sequences of our 13 STEC O104:H4 isolates and a phylogenetic tree was calculated as described in the Materials and Methods section. Using a set of 224 selected SNPs gives rise to the tree shown in Fig 5. Obviously, all epidemic strain isolates including the US-isolate C-3493 which originated from a person who travelled to Germany during the epidemic in 2011, cluster into one clade. The pairwise deviation among them with respect to numbers of SNPs ranges from three to thirty-six (S4 Table). The two German sporadic isolates 11–06681 and 11–07153 which differ from one another by only 10 SNPs do cluster separately from the epidemic strain isolates together with several recent sporadic isolates from France and with historical isolates from Georgia. Moreover, the two historical strains isolated in France in 2004 and 2009, respectively, belong to an even more distant clade. Furthermore, the German historical STEC O104:H4 strain 01–09591 isolated in 2001 is clearly separated from all other isolates.

In the studies by Grad et al. and Guy et al. [22, 30, 31] comparing isolates from the German and the French STEC O104:H4 outbreaks in 2011, a number of SNPs were identified and have been mapped onto the TY2482 chromosome. Accordingly, there are two SNPs that define the German outbreak clade. We extracted the respective positions from the sequence of TY2482 together with surrounding 100 bases upstream and downstream and used the resulting 201-nucleotide sequences to examine the corresponding regions in our genome sequences using the bl2seq-facility at the NCBI BLAST homepage. The two SNPs typical of the German outbreak strain do also occur in all of our outbreak isolates. They are not present, however, in our two sporadic isolates and in the historical strain, which all have a G instead of an A at the position corresponding to 1568661 in TY2482 and a G instead of an A at the position corresponding to 2252380 in TY2482. The same way we could confirm that two SNPs identified as unique to TY2482 by Guy et al. [30] are not present neither in any of our outbreak isolates nor in the two sporadic isolates or in the historical strain. In the analyses performed by Grad et al. and Guy et al. [22, 30] there is only one (from a Swedish who travelled to Germany) among 22 German isolates that was assigned to a clade different from the one where all other German isolates accumulate. For this isolate three SNPs (at positions 1262666, 2564789 and 3089339) were identified. Our two sporadic isolates which would also not fall into the German outbreak clade do not have these SNPs, indicating an independent ancestry of these strains. Moreover, they do not belong to any of the other lineages identified in the French outbreak because none of the 18 SNPs detected in the 11 French outbreak strains studied by Grad et al. [22, 31] is present.

#### Identification and homology analysis of selected features of the sporadic STEC O104:H4 isolates 11–06681 and 11–07153

Based on the contigs assembled for each of the strains, selected features were compared using the BLAST facilities at NCBI. As a quality check of our genome sequences, we analysed the alleles of those housekeeping genes targeted by classical MLST [16, 32] in silico by BLAST analysis of the assembled contigs. Using the sequences determined from the respective PCR products, there was perfect homology confirming the MLST sequence type ST678 (*adk*6, *fumC*6, *gyrB*5, *icd*136, *mdh*9, *purA*7, *recA*7) in all of our isolates.

The Shiga toxin-encoding *E*. *coli* phage P13374 was induced from a German STEC O104:H4 outbreak isolate and sequenced to completion by Beutin et al. [33]. We extracted from the complete prophage genome (accession number HE664024) the *stx*-operon including 200 nucleotides adjacent to each end to give a sequence 1641 nucleotides in length. This sequence was used for BLAST analyses of the contigs assembled for the outbreak isolate 11–02027, for the two sporadic STEC O104:H4 isolates 11–06681 and 11–07153 and for the historical isolate 01–09591 and was found completely present in one of the contigs of each genome sequence, respectively. For 11–02027 and for 01–09591, a sequence identical to *E*. *coli* phage P13374 was observed. Strains 11–06681 and 11–07153, however, show a difference of two nucleotides, one silent T to A mutation at position 909 in the open reading frame of the *stxA*_2a_ subunit gene and another G to A exchange 133 nucleotides downstream of the stop codon of the *stxB*_2a_ subunit gene resulting in an exchange of serine to leucine in a predicted hypothetical protein. Comparing the corresponding *stx*_2_-operon containing DNA segment of 11–07153 with the two Georgian STEC O104:H4 isolates from 2009 as sequenced by Ahmed et al. [21] revealed identity with strain 2009EL-2050. In contrast, the other strain 2009EL-2071 only showed the G to A exchange 133 nucleotides downstream of the stop codon of the *stxB*_2a_ subunit gene whereas the silent mutation in the open reading frame of the *stxA*_2a_ subunit gene was not present.

The respective *stx*-operon-comprising contig from the sporadic strain 11–07153 genome sequence aligns to the phage P13374 sequence continuously along about half of the phage genome to 96.1% pairwise identity. The equivalent contig of 11–06681 contains the left end of the phage and aligns continuously along about 40% of the phage sequence with 95.5% pairwise identity. An appropriate BLAST-based approach, using each of the ends of the prophage and 1000 nucleotides of the adjacent DNA sequence from the TY2482 chromosome, mapped the phage integration site in the genome of isolates 11–07153 and 11–06681 to exactly the same position as in the outbreak strain 11–02027 inside the flavoprotein gene *wrbA*, that is altered due to the integration [33].

Grad et al. [23] discussed the *gyrA* mutations responsible for the nalidixic acid resistance of their O104:H4 strains. They found an amino acid exchange S83A in the GyrA protein sequence of the 2011 epidemic O104:H4 strain as well as in all sporadic isolates from 2011 but a GyrA S83L exchange in HUSEC041, the historical EHEC O104:H4 strain from the HUSEC collection [14] and in their two historical French isolates from 2004 and 2009, respectively. Analysing the genome sequences of the epidemic isolate 11–02027, of the two sporadic isolates 11–06681 and 11–07153 and of the historical strain 01–09591 we found a complete *gyrA* gene assembled into one of the contigs of each of the genome sequences. The predicted GyrA protein sequences of all but strain 01–09591 were identical with an alanine at position 83 of the translated protein sequence revealing the S83A mutation responsible for the Nal phenotype (Table 1). The German historical strain 01–09591 revealed a GyrA S83L exchange as seen in HUSEC041.

In order to identify the chloramphenicol resistance determinant of strain 11–07153 (Table 1), a BLAST analysis in our genome sequence assembly was performed using the open reading frames of common chloramphenicol resistance genes such as *cat* (extracted from BX664015), *cmlA* (AY509004) and *floR* (AF231986) as a query. Not any of these genes was found assembled into a contig of 11–07153. However, mapping of all sequence reads of this genome onto the open reading frames of the chloramphenicol resistance genes identified *floR* but not *cat* or *cmlA* present in 11–07153. For the 11–06681 genome, no homology with any of the chloramphenicol resistance genes was detected, what is in agreement with the chloramphenicol sensitive phenotype of this strain (Table 1). The *floR* gene of 11–07153 differs from the most similar *floR*-allele (accession number AB591424) on a *Salmonella* plasmid by one nucleotide (transversion of A to C) at position 93 in the open reading frame, resulting in an amino acid exchange of leucine for isoleucine.

#### Analysis of the plasmids of the sporadic STEC O104:H4 isolates 11–06681 and 11–07153

We investigated whether the genome sequences of the outbreak index strain isolate 11–02027 and those of the two sporadic strains 11–06681 and 11–07153 contained the DNA sequences of plasmids from other STEC O104:H4 strains by mapping all reads of our genome sequences to the published plasmid reference sequences of four independent STEC O104:H4 isolates as decribed in the Materials and Methods section. The results are compiled in the supplementary file S3 Table. Plasmid profiling revealed that 11–02027 contains three plasmids (Fig 2) corresponding in size to those identified in the outbreak strain reference genomes of TY2428 [8, 9] and 2011C-3493 [21]. Mapping the genome sequence reads of the outbreak strain isolate 11–02027 to each of the plasmids of the outbreak strain reference isolates gave 100% coverage of these plasmids. This is not a direct proof that 11–02027 contains these plasmids, but rather indicates that a complete equivalent of the DNA of these plasmids is also present in 11–02027. However given the results of plasmid profiling (Fig 2), chromosomal SNP analysis (Fig 5) and epidemiological evidence of 11–02027 being an outbreak strain isolate it is reasonable to conclude that the 100% coverage actually indicates presence of the plasmids. By a similar line of arguments one might conclude that the 100% coverage of the EAEC-type adherence-encoding plasmids and of a small cryptic plasmid common to all of the reference genomes in the genome sequences of the two sporadic isolates 11–06681 and 11–07153 does indicate the presence of those plasmids in these strains. The coverage of the ESBL-encoding epidemic reference strain plasmid pTY-1 or pESBL-EA11 in the genome sequences of the two sporadic isolates 11–06681 and 11–07153 is only around 50% (S3 Table). Therefore, one can conclude that such a plasmid is not present in the sporadic isolates what is in agreement with their plasmid profiles (Fig 2) and phenotypes (Table 1). A coverage level of 50% might indicate that the sporadic isolates contained related plasmids (e.g. of the same incompatibility group) which share some plasmid specific sequences encoding similar replication and conjugative transfer systems with pTY-1 and pESBL-EA11. Alternatively there could be some mobile genetic elements (e.g. transposons, IS elements) present somewhere in the genome of the sporadic strains which are also present as accessory genetic elements on pTY-1 and pESBL-EA11. Finally, only a coverage of less than 5% for the large IncF plasmid unique to strain 2009EL-2050 was detected, indicating that there is no similar plasmid present in the German sporadic STEC O104:H4 strains 11–06681 and 11–07153.

## Discussion

Infections with pathogenic *E*. *coli* of the serovar O104:H4 only rarely were reported before the year 2011. An enteroaggregative *E*. *coli* isolated in the 1990s in Africa [34] and a Shiga toxin-producing isolate obtained in 2001 in Germany, HUSEC041 [14], which is identical to the strain 01–09591 included in our investigations, have been the best studied strains of this serovar for many years. After the 2011 outbreaks with a Shiga toxin-producing, enteroaggregative *E*. *coli* O104:H4 in Germany [1] and France [2], substantial effort has been made in studying this emerging pathogen. A few more historical isolates were extensively characterized in comparison to the more recent outbreak strains [21–23, 35, 36]. Whole genome sequencing approaches in addition to conventional typing methods, rapidly uncovered the particular pathogenetic background of this uncommon pathogen [5–11].

Moreover, including numerous additional genome sequences obtained from independent isolates from the German and from the French outbreak, comprehensive SNP analyses provided insight into the phylogenetic relatedness among more recent isolates from 2011 and historical strains [21, 22, 30, 31, 36]. The SNP analyses of several German 2011 outbreak isolates clearly separated them from all historical strains and disclosed only very few differences among them. Even though a slightly greater diversity was observed for the French outbreak strain the degree of conservation among the genome sequences of isolates collected throughout the outbreaks in 2011 [22, 31] was remarkable. The nearly clonal nature of the German isolates in contrast to the more diverse groups of isolates from the much smaller French outbreak led to the hypothesis of a single “major source” for the German outbreak [22, 31] what is in accordance with the conclusions drawn from epidemiological investigations [1]. Our SNP typing results are in good agreement with those of Grad et al. [22, 31] and Guy et al. [30]. The SNP-based tree shown in Fig 5 confirms the phylogenetic relatedness among all of the STEC O104:H4 isolates as suggested by serotyping. However, it also reveals considerable accumulation of mutations during the independent evolution of strains, allowing to discriminate between isolates belonging to the outbreak and epidemiologically obviously unrelated sporadic isolates of this serovar.

Besides the ten outbreak-related STEC O104:H4 isolates, we investigated two distinct STEC O104:H4 strains, 11–06681 and 11–07153, which were isolated in 2011 in Germany and resemble the outbreak strain not only regarding the MLST and the serotype, but also with respect to the virulence gene profile, the *stx*_2_ operon-containing prophage, the expression of Shiga toxin, the degree of cytotoxicity (Fig 3) and the aggregative adherence pattern (Fig 4). However, they differ from the outbreak strain and from each other as well as from the historical 2001 isolate with respect to the antibiograms (Table 1), the PFGE patterns (Fig 1) and the plasmid profiles (Fig 2, S3 Table). Moreover, although isolated in close spatiotemporal proximity to the outbreak in 2011, according to the SNP-based phylogenetic analysis they are clearly separated from outbreak strain isolates (Fig 5). In particular, at the two positions corresponding to 2252380 and 1568661, respectively, in TY2482, which define the clade comprising the German outbreak isolates in the study of Grad et al. [22, 31], they differ from all of the outbreak isolates. Moreover, none of the SNPs identified by Grad et al. [22, 31] and Guy et al. [30] in German or French outbreak isolates is present in neither 11–06681 nor 11–07153 indicating that there is no direct ancestry. Concluding from SNP analyses, the two German sporadic STEC O104:H4 isolates from 2011 are closely related to each other and in terms of phylogeny less distant to the two historical Georgian isolates described by Ahmed et al. [21] than to the historical German isolate from 2001 or to the two historical isolates from France (Fig 5). Moreover, the two sporadic German STEC O104:H4 isolates from 2011 fall into one clade with more recent isolates obtained from sporadic cases in France in 2011 and 2012, respectively (Fig 5).

It is interesting to note, that despite beeing phylogenetically closely related (Fig 5, S4 Table) the two German sporadic STEC O104:H4 isolates considerably differ from each other with respect to their plasmid content. Strains 11–06681 and 11–07153 both do contain a plasmid, identical in size to the EAEC-type adherence-encoding plasmid in the epidemic STEC O104:H4 strain (Fig 2). Most likely, these plasmids are genetically identical or very similar, since the entire DNA sequence of the EAEC-type adherence-encoding plasmid is also present in the genome sequences of strains 11–06681 and 11–07153 (S3 Table **)**. However, there are several more unknown large plasmids in strain 11–07153 and 11–06681 (Fig 2).

Grad et al. [23] concluded from their comprehensive genome comparison analyses that there were two lineages among STEC O104:H4 strains, one comprising the two French historical isolates from 2004 and 2009 and another comprising the 2011 epidemic strain and several French sporadic isolates from 2011 and 2012. These two lineages separated before independently acquiring distinct *gyrA* mutations resulting in resistance to nalidixic acid [23]. In agreement with this conclusion and the SNP-based tree shown in Fig 5, our two German sporadic isolates belong to the lineage comprising the 2011 epidemic strain and the 2011–2012 French sporadic isolates in sharing their GyrA S83A genotype, whereas the German isolate from 2001 shares the GyrA S83L genotype with the French historical isolates. Overall, for the two German sporadic STEC O104:H4 isolates under investigation, the analysis of their whole genome sequences confirms the conclusions drawn from conventional epidemiological subtyping. Moreover, the results do not support a direct derivation of these isolates from the outbreak strain.

The STEC O104:H4 strain Ec11-9450 isolated in October 2011 by Jourdan-da Silva et al. from a French tourist who developed HUS after returning from Turkey revealed a virulence gene profile closely resembling that of the 2011 outbreak strain but the PFGE pattern was slightly different [37]. Moreover, the antibiogram of this strain is similar to that of the 2011 outbreak strain except for the absence of an extended-spectrum betalactamase phenotype, thus resembling the antibiogram of the German sporadic isolate 11–06681 (Table 1). Strain Ec11-9450 was sequenced by Grad et al. and was also found closely related but distinct and not derived from the 2011 outbreak strain [23]. Our SNP-analysis revealed close similarity of the two German sporadic isolates 11–07153 and 11–06681 with Ec11-9450 (Fig 5). In the light of the report of Jourdan-da Silva et al. with respect to the Turkish origin of their STEC O104:H4 isolate it is worth mentioning, that the two German sporadic STEC O104:H4 strains 11–06681 and 11–07153 (Table 1) were isolated from patients with a travel history to Turkey [38]. However, it is not completely without doubt whether the German patients were infected in Turkey because the onset of symptoms or date of isolation, respectively, was only late after returning from Turkey (11 and 18 days, respectively) in contrast to the French traveler, where there is strong epidemiological evidence that this infection was contracted in Turkey [37].

In conclusion, we here describe two additional EHEC/EAEC strains of the serovar O104:H4 which share virulence determinants with the 2011 outbreak strain and with several other sporadic STEC O104:H4 isolates but are epidemiologically unrelated. The results of our investigation of STEC O104:H4 strains add to the knowledge about this emerging pathogen, concerning a certain diversity within the bacterial core genome as well as loss and gain of accessory elements such as plasmids. Our results do also support the view that distinct new variants of STEC O104:H4 might repeatedly originate from a yet unknown reservoir, rather than that there would be a continuous diversification of a single strain established and circulating in Germany after the large outbreak in 2011 [38, 39, 40].

## Supporting Information

S1 Dataset Positions in the TY2482 genome of the 224 SNPs selected for phylogenetic analysis. (TXT)

S2 Dataset Sequences in fasta format created by concatenating those nucleotides from each of the 23 genome sequences under investigation which correspond to the positions of the 224 SNPs selected for phylogenetic analysis (see also S1 Dataset for SNP positions in the TY2482 genome). (FASTA)

S1 Table Sequencing statistics. (DOCX)

S2 Table Coverage of chromosomal coding sequences identified for the reference genome of STEC O104:H4 strain TY2482. (XLSX)

S3 Table Coverage of reference plasmids from various STEC O104:H4 reference genomes. (XLSX)

S4 Table Matrix giving the numbers of SNPs different between the STEC O104:H4 genomes under investigation. (XLSX)

## References

[1] Frank C, Werber D, Cramer JP, Askar M, Faber M, an der Heiden M, et al Epidemic profile of Shiga-toxin-producing *Escherichia coli* O104:H4 outbreak in Germany. N Engl J Med. 2011;365: 1771–1780. 10.1056/NEJMoa1106483 21696328

[2] Gault G, Weill FX, Mariani-Kurkdjian P, Jourdan-da Silva N, King L, Aldabe B, et al Outbreak of haemolytic uraemic syndrome and bloody diarrhoea due to *Escherichia coli* O104:H4, south-west France, June 2011. Euro Surveill. 2011 6 30 16(26). pii: 19905 21749817 10.2807/ese.16.26.19905-en

[3] Mariani-Kurkdjian P, Bingen E, Gault G, Jourdan-Da Silva N, Weill FX. *Escherichia coli* O104:H4 south-west France, June 2011. Lancet Infect Dis. 2011;11: 732–733. 10.1016/S1473-3099(11)70266-3 21958580

[4] Characteristics of the pathogen and information and assistance by the RKI in diagnosis of the currently circulating outbreak strain. Robert Koch-Institute, Germany, Berlin 2011 Available: http://www.rki.de/EN/Home/EHECO104.pdf?__blob=publicationFile. Accessed 2015 Feb 24.

[5] Bielaszewska M, Mellmann A, Zhang W, Köck R, Fruth A, Bauwens A, et al Characterisation of the *Escherichia coli* strain associated with an outbreak of haemolytic uraemic syndrome in Germany, 2011: a microbiological study. Lancet Infect Dis. 2011;11(9): 671–676. 10.1016/S1473-3099(11)70165-7 21703928

[6] Scheutz F, Møller Nielsen E, Frimodt-Møller J, Boisen N, Morabito S, Tozzoli R, et al Characteristics of the enteroaggregative Shiga toxin/verotoxin-producing *Escherichia coli* O104:H4 strain causing the outbreak of haemolytic uraemic syndrome in Germany, May to June 2011. Euro Surveill. 2011 6 16 16(24). pii: 19889 21699770 10.2807/ese.16.24.19889-en

[7] Mellmann A, Harmsen D, Cummings CA, Zentz EB, Leopold SR, Rico A, et al Prospective genomic characterization of the German enterohemorrhagic *Escherichia coli* O104:H4 outbreak by rapid next generation sequencing technology. PLoS One. 2011;6: e22751 10.1371/journal.pone.0022751 21799941 PMC3140518

[8] Rohde H, Qin J, Cui Y, Li D, Loman NJ, Hentschke M, et al Open-source genomic analysis of Shiga-toxin-producing *E*. *coli* O104:H4. N Engl J Med. 2011;365: 718–724. 10.1056/NEJMoa1107643 21793736

[9] Li D, Xi F, Zhao M, Chen W, Cao S, et al Genomic data from *Escherichia coli* O104:H4 isolate TY-2482. BGI Shenzhen 2011 Available: 10.5524/100001 Accessed 2015 Feb 24.

[10] Rasko DA, Webster DR, Sahl JW, Bashir A, Boisen N, Scheutz F, et al Origins of the *E*. *coli* Strain Causing an Outbreak of Hemolytic-Uremic Syndrome in Germany. N Engl J Med. 2011;365: 709–717. 10.1056/NEJMoa1106920 21793740 PMC3168948

[11] Brzuszkiewicz E, Thurmer A, Schuldes J, Leimbach A, Liesegang H, Meyer FD, et al Genome sequence analyses of two isolates from the recent *Escherichia coli* outbreak in Germany reveal the emergence of a new pathotype: Entero-Aggregative-Haemorrhagic *Escherichia coli* (EAHEC). Arch Microbiol. 2011;193: 883–891. 10.1007/s00203-011-0725-6 21713444 PMC3219860

[12] Bakteriologische Untersuchungen im Rahmen des Ausbruchs mit *E*. *coli* O104:H4. Robert Koch-Institute, Germany, Berlin. 2011 Epid Bull. 35: 325–329. Available: https://www.rki.de/DE/Content/Infekt/EpidBull/Archiv/2011/Ausgaben/35_11.pdf?__blob=publicationFile. Accessed 2015 Feb 24.

[13] Report: Final presentation and evaluation of epidemiological findings in the EHEC O104:H4 outbreak, Robert Koch-Institute, Germany, Berlin 2011 Available: http://www.rki.de/EN/Home/EHEC_final_report.pdf?__blob=publicationFile. Accessed 2015 Feb 24.

[14] Mellmann A, Bielaszewska M, Köck R, Friedrich AW, Fruth A, Middendorf B, et al Analysis of a collection of hemolytic uremic syndrome-associated enterohemorrhagic *Escherichia coli* . Emerg Infect Dis. 2008;14: 1287–1290. 10.3201/eid1408.071082 18680658 PMC2600372

[15] Prager R, Fruth A, Busch U, Tietze E. Comparative analysis of virulence genes, genetic diversity, and phylogeny of Shiga toxin 2 g and heat-stable enterotoxin STIa encoding *Escherichia coli* isolates from humans, animals, and environmental sources. Int J Med Microbiol. 2011;301: 181–191. 10.1016/j.ijmm.2010.06.003 20728406

[16] *Escherichia coli* MLST Database [Internet]. Available: http://mlst.warwick.ac.uk/mlst/dbs/Ecoli. Accessed 2015 Feb 24.

[17] Prager R, Lang C, Aurass P, Fruth A, Tietze E, Flieger A, et al Two Novel EHEC/EAEC Hybrid Strains Isolated from Human Infections. PLoS One. 2014;9: e95379 10.1371/journal.pone.0095379 24752200 PMC3994036

[18] Langmead B, Salzberg SL. Fast gapped-read alignment with Bowtie 2. Nat Methods. 2012;9: 357–359. 10.1038/nmeth.1923 22388286 PMC3322381

[19] automatic annotation of the third BGI assembly of the *E* *coli* TY-2482 strain genome. Available: https://github.com/ehec-outbreak-crowdsourced/BGI-data-analysis/wiki/Automatic-annotation-of-bgi-v3-assembly-of-e.-coli-ty-2482-genome. Accessed 2015 Feb 24.

[20] McKenna A, Hanna M, Banks E, Sivachenko A, Cibulskis K, Kernytsky A, et al The Genome Analysis Toolkit: a MapReduce framework for analyzing next-generation DNA sequencing data. Genome Res. 2010;20:1297–1303. 10.1101/gr.107524.110 20644199 PMC2928508

[21] Ahmed SA, Awosika J, Baldwin C, Bishop-Lilly KA, Biswas B, Broomall S, et al Genomic Comparison of *Escherichia coli* O104:H4 Isolates from 2009 and 2011 Reveals Plasmid, and Prophage Heterogeneity, Including Shiga Toxin Encoding Phage stx2. PLoS One. 2012;7: e48228 10.1371/journal.pone.0048228 23133618 PMC3486847

[22] Grad YH, Lipsitch M, Feldgarden M, Arachchi HM, Cerqueira GC, Fitzgerald M, et al Genomic epidemiology of the *Escherichia coli* O104:H4 outbreaks in Europe, 2011. Proc Natl Acad Sci USA. 2012;109: 3065–3070. 10.1073/pnas.1121491109 22315421 PMC3286951

[23] Grad YH, Godfrey P, Cerquiera GC, Mariani-Kurkdjian P, Gouali M, Bingen E, et al Comparative genomics of recent Shiga toxin-producing *Escherichia coli* O104:H4: short-term evolution of an emerging pathogen. mBio. 2013;4: e00452–12. 10.1128/mBio.00452-12 23341549 PMC3551546

[24] Huelsenbeck JP, Ronquist F. MRBAYES: Bayesian inference of phylogeny. Bioinformatics. 2001;17: 754–755. 11524383 10.1093/bioinformatics/17.8.754

[25] Chevreux B. MIRA: An Automated Genome and EST Assembler [dissertation] German Cancer Research Center Heidelberg; 2005 Available: http://www.chevreux.org/thesis/index.html. Accessed 2015 Feb 24.

[26] MIRA web site [Internet]. Available: http://sourceforge.net/projects/mira-assembler. Accessed 2015 Feb 24.

[27] Geneious 7.1 created by Biomatters. Available from http://www.geneious.com. Accessed 2015 Feb 24.

[28] Zhang Z, Schwartz S, Wagner L, Miller W (2000) A greedy algorithm for aligning DNA sequences. J Comput Biol 7: 203–214. 10890397 10.1089/10665270050081478

[29] Basic Local Alignment Search Tool [Internet]. National Center for Biotechnology Information. Available: http://blast.ncbi.nlm.nih.gov/Blast.cgi. Accessed 2015 Feb 24.

[30] Guy L, Jernberg C, Ivarsson S, Hedenström I, Engstrand L, Andersson SG. Genomic diversity of the 2011 European outbreaks of *Escherichia coli* O104:H4. Proc Natl Acad Sci USA. 2012;109: E3627–E3628. 10.1073/pnas.1206246110 23248326 PMC3535599

[31] Grad YH, Lipsitch M, Griggs AD, Haas BJ, Shea TP, McCowan C, et al Reply to Guy et al.: Support for a bottleneck in the 2011 *Escherichia coli* O104:H4 outbreak in Germany. Proc Natl Acad Sci USA. 2012;109: E3629–E3630. 23479789 10.1073/pnas.1209419110 PMC3535640

[32] Wirth T, Falush D, Lan R, Colles F, Mensa P, Wieler LH, et al Sex and virulence in *Escherichia coli*: an evolutionary perspective. Mol Microbiol. 2006;60: 1136–1151. 16689791 10.1111/j.1365-2958.2006.05172.x PMC1557465

[33] Beutin L, Hammerl JA, Strauch E, Reetz J, Dieckmann R, Kelner-Burgos Y, et al Spread of a distinct Stx2-encoding phage prototype among *E*. *coli* O104:H4 strains from outbreaks in Germany, Norway and Georgia. J Virol. 2012;86: 10444–10455. 10.1128/JVI.00986-12 22811533 PMC3457275

[34] Mossoro C, Glaziou P, Yassibanda S, Lan NT, Bekondi C, Minssart P, et al Chronic diarrhea, hemorrhagic colitis, and hemolytic-uremic syndrome associated with HEp-2 adherent *Escherichia coli* in adults infected with human immunodeficiency virus in Bangui, Central African Republic. J Clin Microbiol. 2002;40: 3086–3088. 12149388 10.1128/JCM.40.8.3086-3088.2002 PMC120615

[35] Monecke S, Mariani-Kurkdjian P, Bingen E, Weill FC, Baliere C, Slickers P, et al Presence of Enterohemorrhagic *Escherichia coli* ST678/O104:H4 in France Prior to 2011. Appl Environ Microbiol. 2011;77: 8784–8786. 10.1128/AEM.06524-11 22003010 PMC3233108

[36] Guy L, Jernberg C, Arvén Norling J, Ivarsson S, Hedenström I, Melefors Ö, et al Adaptive Mutations and Replacements of Virulence Traits in the *Escherichia coli* O104:H4 Outbreak Population. PLoS One. 2013;8(5): e63027 10.1371/journal.pone.0063027 23675451 PMC3651199

[37] Jourdan-da Silva N, Watrin M, Weill FX, King LA, Gouali M, Mailles A, et al Outbreak of haemolytic uraemic syndrome due to Shiga toxin-producing *Escherichia coli* O104:H4 among French tourists returning from Turkey, September 2011. Euro Surveill. 2012 1 26 17(4). pii: 20065 22297137 10.2807/ese.17.04.20065-en

[38] Frank C, Milde-Busch A, Werber D. Results of surveillance for infections with Shiga toxin-producing *Escherichia coli* (STEC) of serotype O104:H4 after the large outbreak in Germany, July to December 2011. Euro Surveill. 2014 4 10 19(14). pii: 20760 24739983 10.2807/1560-7917.es2014.19.14.20760

[39] Abu Sin M, Takla A, Flieger A, Prager R, Fruth A, Tietze E, et al Carrier prevalence, secondary household transmission and long-term shedding in two districts during the *Escherichia coli* O104:H4 outbreak in Germany, 2011. J Infect Dis. 2013;207:432–438. 10.1093/infdis/jis702 23175763

[40] De Rauw K, Vincken S, Garabedian L, Levtchenko E, Hubloue I, Verhaegen J, et al Enteroaggregative Shiga toxin-producing *Escherichia coli* of serotype O104:H4 in Belgium and Luxembourg. New Microbes New Infect. 2014;2(5): 138–143. 10.1002/nmi2.58 25356363 PMC4184478

