# Supplementary material for: Comparative Genomic Analysis of Two Novel Sporadic Shiga Toxin-Producing Escherichia coli O104:H4 Strains Isolated 2011 in Germany
Source: PLoS One. 2015 Apr 2;10(4):e0122074. doi: 10.1371/journal.pone.0122074 (PMC4383531; doi:10.1371/journal.pone.0122074)
Supplement: S1 Table — (DOCX) [file pone.0122074.s003.docx]

**Table S1**. Sequencing statistics

| **Strain** | **mapping coverage of the TY2482-chromosome** | **No. of reads** | **No. of bases** | **average read length** |
| --- | --- | --- | --- | --- |
| 11-02027 | 99.22 | 805425 | 461279220 | 573 |
| 11-06681 | 98.21 | 933170 | 539763994 | 578 |
| 11-07153 | 98.18 | 615219 | 334931392 | 544 |
| 11-02058 | 99.17 | 220504 | 150563205 | 683 |
| 11-02135 | 99.04 | 87815 | 59555589 | 678 |
| 11-03424 | 99.22 | 286880 | 194094383 | 677 |
| 11-03944 | 99.17 | 208897 | 142221879 | 681 |
| 11-04083 | 99.23 | 337342 | 230240744 | 683 |
| 11-06811 | 96.79 | 117963 | 29287536 | 248 |
| 11-06837 | 95.07 | 91008 | 22588919 | 248 |
| 01-09591 | 96.4 | 212098 | 51079226 | 241 |
| 11-06601 | 97.02 | 116291 | 29806527 | 256 |
| 11-04782 | 98.35 | 160742 | 43477617 | 270 |
